# Monthly Summary

**Published:** 1883-07

**Authors:** 


### Monthly Summary.

Inebriety and the Teeth.—Dr. T. O. Crothers writes to the *Medical Journal* as follows: In a paragraph with this heading you refer to the experience of a clergyman, who thinks he has seen less drunkenness in cases where the teeth have been treated and properly cared for.

This is the recognition of a fact which appears very often in the history of cases of inebriety, namely, the presence of distinct physical causes which are both exciting and predisposing in every case. As, for instance, a man of eminence will invariably drink to intoxication, after a hearty dinner at midnight. Avoiding this, he is abstinent and can fully control himself. A business man never uses spirits to excess, except when he sits up all night or travels by rail. After this exposure he is powerless to stop short of profound intoxication. With proper rest every night he can be a thorough temperance man. A broker was forced to retire from business because of excessive use of spirits. He was able to fully abstain as a manufacturer in a quiet town. Every time he went back to Wall Street, even for a few hours, he drank, although not tempted in any way more than others.

In the *Journal of Inebriety*, vol. ii., Dr. Harman, of Ohio, reports a case of a pronounced inebriate who recovered, and remained a sober man ever after, dating from the expulsion of a tape-worm. An officer in the late war, who was considered a chronic inebriate, dating from a wound of the tibia, recovered immediately after the removal of some dead bone and the healing of the wound. He had tried repeatedly before to abstain, but failed. The recovery after the operation was in circumstances more adverse than ever before. The late Dr. March, of Albany, trephined the skull of a man who had drank to great excess, from the time of an injury by a fall on the head,

The man recovered and never used spirits after, for a period of eight years, up to his death.

In an article in the *Chicago Medical Journal* for November, 1881, I have stated many of these singular cases, where injury and irritation of any part of the body may react by some unknown law and develop inebriety. In many of the cases which come under my care there is often appearently very insignificant states of the body, which are found to be prominent in the causation—sources of irritation and exhaustion, neuralgias, nutrient disturbances, and local derangements of almost every description, the removal of which is followed by a rapid cessation of the desire for drink, and the cure of inebriety.

The teeth may very naturally be sources of irritation, which if it does not cause inebriety, will most naturally keep up the irritation which provokes a continuance of this disorder.

In the majority of thesc cases a special diathesia may be the favoring soil, which will develop inebriety from the slightest. A neurasthenic state and general nerve instability, for which alcohol is a most seductive sedative, and inebriety follows with great certainty.

It is only a rational expectation to find that decayed teeth was an exciting cause, and inebriety would be more manageable when this source of irritation was removed. Recovery cannot be expected until all sources of irritation can be more or less removed. The clergyman who insisted on the care and treatment of the teeth in inebriety as a part of the treatment, was following the teachings of the most advanced science of to-day. If in addition, nutrition, surroundings, and the removal of all exciting causes was made a part of the treatment, recovery would be the rule and failure the exception. Inebriety is always the result of physical conditions, whether understood or not. The sooner this is recognized and practical treatment based on it, the whole subject will be raised from the realm of superstition and quackery.

The curability of inebriety by physical means and remedies is as practical and real as that of any other disorder. What is wanted is a thorough study of the subject from the standpoint of science, above all theories and dogmas of to-day.

TO STOP HICCOUGH.—Dr. Martin Burke, of this city, sends us the following item: "Perhaps the narrative of these two cases may prove of interest, John C—— was suddenly seized about a year ago with an attack of hiccough. The cause was unknown. All the usual remedies were tried in vain. Dr. John Burke, my father was then called upon. Noticing the convulsive heaving of the patient's ribs, more particularly upon the left side, he firmly compressed the side between his two hands, and in a short time the hiccough ceased for the first time in days. The second case was that of Mr. C——, a young man then with hiccough, most violent and convulsive. Morphine suppositories would produce sleep, but even in sleep, the hiccough was distressingly severe. As his vomiting had now ceased, almost every remedy known was called to our aid but it was not until we had again, by my father's advice, compressed his heaving ribs, that the hiccough almost instantly ceased. It returned indeed within tweenty-four hours, but compression again arrested it. The patient is now convalescing, and as hiccough very often proves fatal, perhaps the record of these two cases may prove of service."—*Medical Record.*

---

TO DISGUISE THE ODOR OF IODOFORM.—Dr. J. A. Andrews, of Staten Island, N. Y., writes: "It may interest your readers to know that cumurin will disguise the unpleasant odor of iodoform. The Tonka bean has been employed for this purpose. This bean being placed in the bottle containing iodoform. This, however, is not effectual. Cumurin, itself a derivative of the Tonka bean, is an anhydrate of cumuric acid. It disguise the odor of iodoform more effectually and permanently than do other drugs with which we are familiar, nor does it form, when incorporated with iodoform, lumps of powder which are slow to dissolve, which is a real advantage where this drug is employed in therapeusis of purulent inflammation of the middle ear. However, it is not the object of my communication to urge the claims of iodoform in the treatment of supperative inflammation of the external or middle ear, because I believe it is of use only in certain selected cases of this affection, often actually aggravating the condition in others. The minimum amount of cumurin which I have found sufficient to disguise the odor of iodoform, is three grains of the former to one drachm of the latter."—*Medical Record.*

---

HOW TO KEEP COOL, has been solved, according to Patent Office report, by a Davenport (Iowa) man, who has perfected a fanning machine for private houses and which is applible to dental offices. The novelty of the thing consists in the fan being confined in a circular metal casing, having four short open pipes protuding from it, top and bottom. This casin is suspended from the ceiling, and inside of it is the fan, revolved by a bar extending the length of the apartment, and connecting with anything at all that will give it motion. The fan makes such a disturbance in the air, through the agency of the distributing pipes, that the atmosphere doesn't have an opportunity of settling right down to the business of getting hot, and so the room is kept cool. If a small edition of this apparatus could be devised for use in hats to keep some excitable heads cool, it would be a brilliant success.—*Decorator and Furnisher.*

---

GLUTEN.—Flour deprived of the *gluten* of the wheat, under which general name may be classed the phosphatic and nitrogenous elements—which are stored principally between the outer wraps and the inner starch body of the kernel—has lost the greater part of its blood-making materials. This gluten of wheat may be compared ro the lean of meat, while the whiter starchy portions of the wheat may be compared to the fat of meat.—*American Miller.*

